# Assessing the impact of community-based interventions on hypertension and diabetes management in three Minnesota communities: Findings from the prospective evaluation of US HealthRise programs

**DOI:** 10.1371/journal.pone.0279230

**Published:** 2023-02-27

**Authors:** Nancy Fullman, Krycia Cowling, Luisa S. Flor, Shelley Wilson, Paurvi Bhatt, Miranda F. Bryant, Joseph N. Camarda, Danny V. Colombara, Jessica Daly, Rose K. Gabert, Katie Panhorst Harris, Casey K. Johanns, Charlie Mandile, Susan Marshall, Claire R. McNellan, Vasudha Mulakaluri, Bryan K. Phillips, Marissa B. Reitsma, Naomi Sadighi, Tsega Tamene, Blake Thomson, Alexandra Wollum, Emmanuela Gakidou

**Affiliations:** 1 Institute for Health Metrics and Evaluation, Department of Health Metrics Sciences, University of Washington, Seattle, Washington, United States of America; 2 US Department of Health and Human Services, Washington, DC, United States of America; 3 Medtronic Foundation, Minneapolis, Minnesota, United States of America; 4 Public Health Seattle and King County, Seattle, Washington, United States of America; 5 University of Washington School of Medicine, University of Washington, Seattle, Washington, United States of America; 6 HealthFinders Collaborative, Northfield, Minnesota, United States of America; 7 International School of Djibouti–Ministere de l’Education Nationale, Djibouti City, Djibouti; 8 School of Social Work, University of North Carolina at Chapel Hill, Chapel Hill, North Carolina, United States of America; 9 The Permanente Medical Group, Oakland, California, United States of America; 10 Small Sums, St Paul, Minnesota, United States of America; 11 Pillsbury United Communities, Minneapolis, Minnesota, United States of America; 12 American Cancer Society, Atlanta, Georgia, United States of America; 13 Ibis Reproductive Health, Cambridge, Massachusetts, United States of America; Kurume University School of Medicine, JAPAN

## Abstract

**Background:**

Community-based health interventions are increasingly viewed as models of care that can bridge healthcare gaps experienced by underserved communities in the United States (US). With this study, we sought to assess the impact of such interventions, as implemented through the US HealthRise program, on hypertension and diabetes among underserved communities in Hennepin, Ramsey, and Rice Counties, Minnesota.

**Methods and findings:**

HealthRise patient data from June 2016 to October 2018 were assessed relative to comparison patients in a difference-in-difference analysis, quantifying program impact on reducing systolic blood pressure (SBP) and hemoglobin A1c, as well as meeting clinical targets (< 140 mmHg for hypertension, < 8% Al1c for diabetes), beyond routine care. For hypertension, HealthRise participation was associated with SBP reductions in Rice (6.9 mmHg [95% confidence interval: 0.9–12.9]) and higher clinical target achievement in Hennepin (27.3 percentage-points [9.8–44.9]) and Rice (17.1 percentage-points [0.9 to 33.3]). For diabetes, HealthRise was associated with A1c decreases in Ramsey (1.3 [0.4–2.2]). Qualitative data showed the value of home visits alongside clinic-based services; however, challenges remained, including community health worker retention and program sustainability.

**Conclusions:**

HealthRise participation had positive effects on improving hypertension and diabetes outcomes at some sites. While community-based health programs can help bridge healthcare gaps, they alone cannot fully address structural inequalities experienced by many underserved communities.

## Introduction

Longstanding health disparities occur throughout the US [1], with differences often manifesting across multiple environmental and societal factors (e.g., geography, sex or gender, race or ethnicity, socioeconomic status) [2]. Non-communicable diseases (NCDs) and NCD-related risks like hypertension can uniquely affect underserved communities, as a constellation of structural factors–from service affordability and inadequate insurance to more entrenched socioeconomic obstacles like low access to nutritional food–can easily give way to high rates of chronic, debilitating conditions experienced in settings without good access to care or appropriate services. Community-based interventions with integrated care have emerged as approaches to bridge gaps in NCD care, particularly for underserved areas in the US [3–9]. Nonetheless, the effectiveness of these interventions vary across contexts; interventions implemented; roles of community health workers (CHWs)(e.g., direct involvement in patient care [10] *versus* assisting health providers who are then responsible for service provision [11]); and NCDs targeted. While CHWs and community-based interventions appear to be promising models of care for hypertension and diabetes in underserved communities [5–7], more rigorous evaluations across more local contexts and populations are needed.

To strengthen this evidence base, the HealthRise program was developed to implement and pilot locally-tailored programs for improving screening, diagnosis, management and control of hypertension and diabetes among underserved communities [12–14]. HealthRise took place in nine communities in four countries–Brazil, India, South Africa, and the US–from 2014 to 2018. In the US, Minnesota was selected, a state which generally surpasses national averages and ranks among the healthiest across many health measures [1, 15]. Nonetheless, county- and sub-county level health disparities remain in Minnesota, particularly among populations that face compounding barriers to care and improved health outcomes [15, 16]. To maximize the potential impact of HealthRise programs, especially within a relatively short time span (i.e., the earliest US program began in 2016), HealthRise targeted geographic areas with the greatest need and highest disease burden. A 2014–2015 needs assessment identified communities within three Minnesota counties–Hennepin, Ramsey, and Rice–as potential candidates for HealthRise programs based on a combination of quantitative indicators and key informant interviews [15, 17]. Such data pointed to high NCD burdens and risk profiles; widespread access challenges for healthcare, education, and nutritious foods; and sociodemographic characteristics often associated with greater healthcare barriers and worse outcomes stemming from structural racism (e.g., large proportions of populations identifying as Hispanic or Latinx, Black or African American; as well as immigrants or refugees). Three main recommendations emerged for onward HealthRise programming: (1) focus on people with the highest need and poorest clinical outcomes (2) use multi-faceted interventions to address multiple risks and comorbid conditions; and (3) identify opportunities to integrate CHWs within formal health system functions [15]. Drawing from the needs assessment and HealthRise grantee applications, three implementing partners were selected for each county–Pillsbury United Communities (PUC) in Hennepin, Regions Hospital Foundation in Ramsey, and HealthFinders Collaborative, Inc (HFC) in Rice–and HealthRise grantees developed their locally tailored community-based interventions and corresponding activities (as summarized Table 1) [14]. US HealthRise program implementation then took place from June 2016 to October 2018.

**Table 1:** Overview of interventions by US HealthRise site. More detailed descriptions of HealthRise interventions, as provided by grantees and compiled by Abt Associates, are available elsewhere [14]. HealthRise grantees implemented community-based interventions and activities associated with each site.

		Hennepin County	Ramsey County	Rice County
**HealthRise grantee and/or local partners**		Pillsbury United Communities (PUC)	Regions Hospital Foundation	HealthFinders Collaborative, Inc (HFC)
		North Rising partnership comprised of PUC and North Memorial Health, a network of hospitals and clinics	Minnesota Community Care (MCC) (formerly named West Side Community Health Services, abbreviated WSCHS)	
**HealthRise implementation location**		North Minneapolis, Minnesota	Saint Paul, Minnesota	Northfield, Minnesota
**HealthRise program period**		July 2016 to September 2018	June 2016 to September 2018	September 2016 to October 2018
**Key characteristics and/or challenges for communities served by HealthRise**	Shared across sites	• **High levels of poverty and/or unemployment** (e.g., 40% at or below 200% poverty line in Hennepin; 97% of MCC clinic patients in Saint Paul are below 200% poverty line)		
		• **High proportion of population are non-white and/or immigrants or refugees** (e.g., past HFC patients were 60% Latino immigrants and 25% Somali refugees in Rice County; 30–65% of MCC clinic patients do not speak English as their primary language; North Minneapolis population is about 50% African American, 20% Asian, 15% Caucasian, and 15% Hispanic/Other)		
		• **Insufficient support of disease management and behavioral changes to improve health within formal health system structures or communities** (e.g., poor access to high-quality education, healthcare, and nutritious foods in Hennepin County; HFC specifically targeted uninsured or individuals with public insurance plans in Rice County)		
		**• Inadequate or poor integration of community health care systems, including data systems** (e.g., minimal integration of electronic health records across community health care sites in Hennepin; data systems between MCC clinics and hospitals in Ramsey were not integrated)		
	Site-specific	• Urban setting (Twin Cities area)	• Urban setting (Twin Cities area)	• Primarily rural
		• Reported low trust in local health systems	• Reported high levels of emergency department repeat users	• Reported high cultural and language barriers among immigrants and undocumented migrant worker population
**Key HealthRise interventions and activities**	Shared across sites	**• Community-based programs and training:** hired and trained community health workers (CHWs) and community paramedics (CPs) to provide home-based care and linkages to clinic-based provider teams (e.g., doctors, nurses, pharmacists, clinical care coordinators, and diabetes education in Hennepin County); for Rice County, where HFC already had pre-existing CHW/CP care teams and networks, additional training and expanded services occurred (e.g., mental health, on-site lab for easier access to diagnostic tests)		
		**• Home-based care:** CHWs and CPs visited patients for disease management (i.e., monitor health status, medications), health education (e.g., health education, healthy food cooking demonstrations), and support for social needs or social determinants of health (e.g., insurance, housing, transportation); often tailored frequency of in-home visits to patient care plans and based on trends in clinical targets		
		**• Technologies for care coordination:** implemented tools to better coordinate care between CHW/CP teams and clinic-based teams (e.g., Pathways from Care Coordination Systems for Ramsey County) or incorporated home visit information into electronic medical record (EMR) systems (e.g., HFC designed EMRs to include in-home information into patient medical records at clinics)		
		**• Community activities and wellness programs:** led via CHWs or supported via community centers to support nutrition education and resource connection (e.g., healthy eating demonstrations in Hennepin County and Opportunity grant nutrition-focused program in Ramsey County); disease management tailored to cultural and linguistic needs (e.g., monthly and quarterly diabetes management classes and Somali Health series in Rice County); and exercise/wellness programs (e.g., Pura Vida which included exercise classes, cooking and nutrition classes, etc. in Rice County).		
	Site-specific	• **Established community-care teams of CHWs and CPs linked to clinic-based care teams:** this model of care was relatively new to PUC and Hennepin County partners, so recruitment of CHWs/CPs and training with care teams occurred alongside other HealthRise-supported activities	• **Established community-care teams of CHWs and CPs linked to clinic-based care teams:** this model of care was relatively new to Regions and Ramsey County partners, so recruitment of CHWs/CPs and training with care teams occurred alongside other HealthRise-supported activities	**• Developed new partnerships to expand community-based care:** partnered with Northfield Hospital and Clinics to expand CP program; collaborating with the Mayo Clinic and Alaian Health System to extend model beyond NCDs (e.g., ob/gym care for Somali populations)
		• **Implemented** i**nterdisciplinary approaches to improving health**: established a full-service grocery store (North Market) with linkages to an interdisciplinary wellness team (e.g., CHWs, nutritionist, pharmacy liaison, coordinator) and a Wellness Resource Center with North Memorial Health	• **Focused on community-based nutrition programs:** used Opportunity Grant to develop and implement a nutrition-focused program, in both English and Spanish, wherein sessions focused on nutrition education, effects of non-nutrition factors on blood sugar (e.g., physical activity, stress management), and grocery store tours highlighting ways to shop for healthy and affordable foods	**• Employed several electronic tools for improving contact with patients:** developed SMS/text-based appointment reminders and education programs (i.e., Care Message)

With this study, we provide key findings from HealthRise programs implemented by grantees in Hennepin, Ramsey, and Rice Counties, Minnesota. Based on quantitative and qualitative data collected over the course of program implementation, we evaluated the potential impact of these community-based interventions on improving clinical and health outcomes for hypertension and diabetes patients. We conducted difference-in-difference analyses in relation to comparison patients to quantify this impact above and beyond what might be expected for demographically similar patients under routine care in the same communities. This study contributes to the science supporting the role of community-based programs in elevating the health of underserved communities, in the US and elsewhere.

## Materials and methods

### Study overview, design, and interventions

This analysis follows the global HealthRise prospective evaluation framework, which was established in 2014 and agreed upon by all partners; greater detail on the global team structure, interventions, and analyses are provided elsewhere [12–14]. In sum, the HealthRise program had global and US implementation partners coordinated by Abt Associates and evaluation activities overseen by the Institute for Health Metrics and Evaluation (IHME). Though ongoing coordination and collaboration occurred across implementation and evaluation organizations, these grant streams were purposefully structured and funded separately to support an independent assessment of the HealthRise programs.

For US HealthRise, patient-level monitoring data were routinely collected and collated by grantees during program implementation (June 2016 to October 2018). Using a mixed-methods quasi-experimental design, we synthesized qualitative and quantitative data from HealthRise and comparison patients and stakeholders (e.g., service providers, administrators, and policymakers) to inform its endline evaluation.

Table 1 summarizes key information on each US HealthRise site and interventions implemented by grantee, as interventions were tailored to address key challenges or structural drivers of inequalities identified for each site during the 2014–2015 needs assessment [15, 17]. Per the needs assessment [15, 17], populations across sites experienced high levels of poverty and/or unemployment; the majority of populations identified as Hispanic or Latinx, Black or African American, as well as immigrants or refugees; and substantive challenges around access to sufficient disease management and health promotion support occurred within formal health system structures and broader communities. HealthRise programming was designed to address both cross-cutting and site-specific needs or challenges; additional descriptions for each site’s interventions and activities as part of HealthRise are available elsewhere [14].

### Definitions and data

#### Definitions

We used following case definitions for hypertension and diabetes at each time point: (1) prevalent cases were patients with documented diagnoses, or patients without prior diagnoses but with clinical readings that would qualify for diagnosis (i.e., systolic blood pressure [SBP] ≥ 140 mmHg or diastolic blood pressure [DBP] ≥ 90 mmHg for hypertension; hemoglobin A1c ≥ 6.5% for diabetes); (2) diagnosed cases were patients with documented diagnoses; and (3) patients meeting treatment targets were prevalent cases with SBP < 140 mmHg and DBP < 90 mmHg for hypertension, and A1c < 8% for diabetes. If DBP measures were not available for a given patient, then only SBP readings were used.

#### Endline evaluation data collection

*HealthRise patient data*. Each US grantee collected patient-level data from existing sources and provided de-identified data over time. To best capture potential program impact, analyses were limited to HealthRise patients who (1) remained enrolled in HealthRise at endline (i.e., never withdrew from programs); and (2) had at least two separate biometric data points for blood pressure (i.e., ideally both SBP and DBP, but at minimum, SBP) or A1c (Table 2). Subsequently, evaluation results reflected potential effects from HealthRise participation, and not “intention to treat,” which would have included patients who enrolled but then withdrew from the program at some point. Rates of any program withdrawal varied by site, ranging from 16.7% (n = 19) for Hennepin to 32.5% (n = 25) for Ramsey. For the Rice HealthRise site, 3.2% of patients (n = 5) lacked a clinic visit or biometric data since baseline, and thus were considered withdrawn. In Ramsey, most patients who withdrew did so after a few months of enrollment and within the first year of HealthRise implementation.

**Table 2:** Endline data availability and patient sample sizes by HealthRise site and for intervention and comparison patients.

Data collection	Hennepin County		Ramsey County		Rice County	
	HealthRise	Comparison	HealthRise	Comparison	HealthRise	Comparison
**Quantitative**						
**Total patients**	121	135	78	104	217	311
Patients with baseline data and meeting inclusion criteria	114	113	77	95	157	311
**Patients enrolled at endline**	**95**	**113**	**52**	**95**	**152**	**311**
Patients with hypertension	85	83	32	66	84	182
**Patients with hypertension and ≥ 2 biometric readings**	**80**	**83**	**32**	**66**	**80**	**170**
Patients with diabetes	76	75	48	78	125	303
**Patients with diabetes and ≥ 2 biometric readings**	**37**	**39**	**43**	**72**	**96**	**296**
**Qualitative**						
**Total interviews and focus groups**	5	-	9	-	6	-
Community health workers and frontline health workers	2	-	3	-	2	-
Facility- or clinic-based providers	1	-	3	-	2	-
Facility or clinic managers and administrators	2	-	3	-	2	-
Policymakers	3					

“Total patients” reflect the number of patients included in original samples for each site’s HealthRise program, irrespective of additional inclusion or exclusion criteria. “Patients with baseline data and meeting inclusion criteria” reflect the number of patients who met inclusion criteria at baseline (i.e, prevalent case of hypertension or diabetes and aged 30–89 years old); for HealthRise patients, this also reflects the number of patients who formally enrolled in the program, whereas comparison patients were limited to individuals who could have met eligibility requirements at each site within the program implementation time periods (2016–2018). “Total patients enrolled at endline” reflect the number of patients who did not withdraw from HealthRise during program duration. At each site and group, some proportion of patients have both hypertension and diabetes.

For baseline measures, we used biometric data collected at HealthRise program enrollment. If such data were not available at the precise enrollment date, then biometric data were used from the data closest to that of enrollment. For endline measures, we used patients’ most recent biometric measurements.

S1 Table provides additional descriptive statistics, including counts and percentages by age, reported sex, and self-identified race or ethnicity, for HealthRise patients at baseline and for those who remained enrolled through endline. Due to already small sample sizes for each site’s HealthRise program and concerns about potential differences or inconsistencies in race or ethnicity response options available across data sources, further analysis disaggregated by age, reported sex, and reported race or ethnicity was not conducted.

*Comparison patient data*. Grantees provided comparison data drawn from patient populations similar to those enrolled in HealthRise. Upon receiving each site’s dataset, we sought to reconstruct samples of comparison patients that were similar demographically and in terms of baseline health conditions to HealthRise patients (i.e., excluding comparison patients younger than 30 years and 90 years or older, and those without a diagnosis of hypertension or diabetes and had baseline biometric data that fell within disease control categories). As necessary, comparison patient data were censored to correspond with each site’s HealthRise program implementation period (Table 1), and thus better approximate similar follow-up times for comparison patients. After this censoring step, we then excluded any comparison patients who lacked more than one measurement of A1c and systolic blood pressure and therefore could not contribute to baseline *versus* endline comparisons. Included comparison patients, by site, are provided in Table 2.

Comparison patient data selection occurred between October 2018 and January 2019, with criteria determined by each HealthRise site. For Hennepin, data for patients who formed the comparison group were extracted from clinics associated with North Memorial but had not enrolled in HealthRise. Selection criteria included having at least two biometric readings for A1c or SBP–one in 2016 and one in 2018 –to approximate baseline and endline measures for HealthRise; and being between the ages of 30 and 89 years at “baseline.” For Ramsey, comparison patient data were extracted through MCC (formerly named West Side Community Health Services); eligible individuals were patients who had not enrolled in HealthRise and had similar baseline levels of A1c or SBP as HealthRise patients. For Rice, data were extracted from a partner clinic where HealthRise interventions were not implemented. Unlike other comparison patient datasets, International Classification of Disease codes for diabetes and hypertension were not available for patient diagnosis; instead, the diagnosis variable for Rice comparison patients listed active diagnoses. Consequently, a text-matching algorithm was applied to assign diabetes and/or hypertension diagnosis based on the text data in this variable.

*Qualitative data*. Twenty-three key informant interviews (KIIs) were conducted with local policymakers (non-site specific) and with different types of staff at each site (Table 2). Interviews were not conducted with patients or with staff at clinics from which comparison patient data were selected.

Initial potential interviewees were identified via leadership from HealthRise grantees and partner organizations, and then additional staff (e.g., clinic-based providers, community paramedics [CPs], CHWs) were contacted via snowball sampling. Of the original individuals identified, 79% completed one-hour interviews via telephone with an IHME evaluation team member.

All interviews were audio-recorded and listened to multiple times by a single researcher. Key components were transcribed in an Excel template, with thematic coding applied to identify both site-specific and overarching themes across sites.

#### Endline evaluation analyses

To quantify potential effects of HealthRise participation, we used two outcome indicators to measure patient-level changes from baseline to endline: (1) the proportion of patients meeting treatment targets (i.e., SBP < 140 mmHg and DBP < 90 mmHg for hypertension; < 8% A1c for diabetes); and (2) patient biometric measures (i.e., SBP for hypertension, A1c for diabetes). All analyses were limited to patients who were prevalent cases at baseline and had corresponding biometric data for each time point.

We conducted difference-in-difference analyses in two steps for each site and by condition. First, we ran an unadjusted model, only including binary variables for HealthRise status and timing (i.e., baseline or endline) and an interaction term for HealthRise at endline to capture the effect of HealthRise participation over time. We then ran an adjusted model, including the following covariates to account for potential systematic differences in HealthRise and comparison patients: sex (female, male); age (< 50 years, ≥ 50 years); time elapsed from baseline to endline (< 12 months, ≥ 12 months); and comorbidities at baseline (prevalent case of only hypertension or diabetes; prevalent case of both hypertension and diabetes). We specified robust standard errors for each model, and used Welch’s t-tests (i.e., assuming unequal variance between each group) to evaluate statistically significant differences between HealthRise and comparison patients. All analyses were conducted in Stata version 15 and R version 3.6.2 [18, 19].

### Ethical approval

Ethical approval for this study was obtained from the University of Washington’s institutional review board, as well as the local data collection agencies and government entities for each site. All personal identifiers were removed prior to data sharing with IHME; only de-identified data were analyzed.

## Results

### Quantitative results

**Fig 1:**
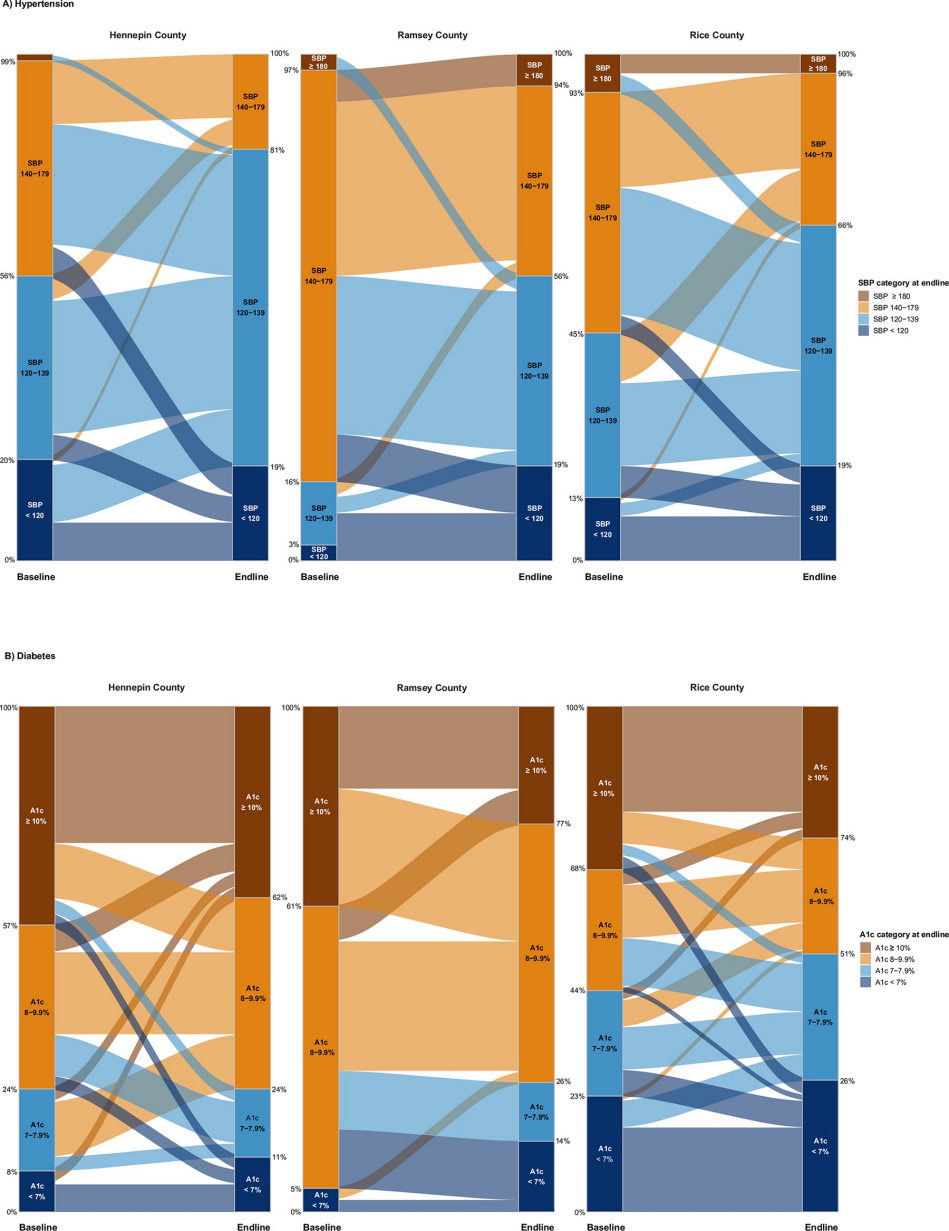
HealthRise patient shifts in disease severity categories between baseline and endline based on biometric readings for hypertension (A) and diabetes (B). The height of each column reflects 100% of patients at each time point (baseline and endline), while the categories within each column represents the percentage of patients in each category at baseline and endline. Patient groups are color-coded by their categorization at endline (right column per site) and flow from their categorization at baseline (left column per site). By category percentages, for each site, are available in S1 Data.

Overall, hypertension and diabetes indicators generally improved for HealthRise patients compared with their baseline measures (Fig 1, Table 3). Yet clinical improvements were heterogeneous since program enrollment (Fig 1), emphasizing potential challenges in effective case management among underserved populations.

**Table 3:** Baseline and endline measures by HealthRise site and for intervention and comparison patients for hypertension (A) and diabetes (B). For HealthRise, samples reflect patients who were prevalent cases of hypertension or diabetes at baseline; remained enrolled throughout the program; and had at least two biometric readings during program participation.

A) Hypertension							
**HealthRise program site**	**Sample size**	**Percent of patients meeting treatment targets (SBP < 140 and DBP < 90) (%)**		**Average SBP reading (mmHg)**		**Average changes in SBP from baseline to endline**	
		**Baseline**	**Endline**	**Baseline**	**Endline**	**Absolute change (mmHg)**	**Percent change (%)**
**Hennepin County**							
HealthRise patients	80	53.8 (42.7 to 64.5)	76.3 (65.6 to 84.4)	136.2 (131.8 to 140.6)	128.5 (125.6 to 131.5)	-7.7 (-12.1 to -3.2)	-4.2 (-7.2 to -1.1)
Comparison patients	83	83.1 (73.3 to 89.8)	78.3 (68.0 to 86.0)	127.3 (124.3 to 130.3)	125.3 (121.9 to 128.7)	-2.0 (-6.0 to 2.0)	-0.7 (-3.9 to 2.5)
**Ramsey County**							
HealthRise patients	32	12.5 (4.6 to 29.8)	50.0 (32.7 to 67.3)	150.4 (144.9 to 156.0)	136.0 (126.6 to 145.5)	-14.4 (-23.0 to -5.8)	-9.4 (-15.0 to -3.9)
Comparison patients	66	30.3 (20.3 to 42.6)	45.5 (33.7 to 57.7)	146.5 (141.5 to 151.6)	142.9 (137.4 to 148.3)	-3.7 (-10.2 to 2.9)	-0.9 (-5.5 to 3.6)
**Rice County**	** **	** **	** **	** **	** **	** **	** **
HealthRise patients	80	42.5 (32.0 to 53.7)	63.8 (52.5 to 73.6)	142.5 (137.5 to 147.6)	134.6 (129.9 to 139.3)	-7.9 (-13.4 to -2.5)	-4.0 (-7.7 to -0.4)
Comparison patients	170	78.2 (71.4 to 83.8)	82.4 (75.8 to 87.4)	128.3 (126.0 to 130.6)	127.3 (124.9 to 129.7)	-1.0 (-3.6 to 1.5)	0.0 (-2.0 to 2.1)
**B) Diabetes**							
**HealthRise program site**	**Sample size**	**Percent of patients meeting treatment targets (A1c < 8%)**		**Average A1c reading (%)**		**Average changes in A1c from baseline to endline**	
		**Baseline**	**Endline**	**Baseline**	**Endline**	**Absolute change (%)**	**Percent change (%)**
**Hennepin County**							
HealthRise patients	37	24.3 (12.9 to 41.1)	24.3 (12.9 to 41.1)	9.9 (9.1 to 10.7)	9.4 (8.6 to 10.3)	-0.5 (-1.4 to 0.5)	-0.8 (-12.6 to 11.1)
Comparison patients	39	71.8 (55.8 to 84.0)	76.9 (60.7 to 87.8)	7.6 (7.1 to 8.2)	7.5 (6.9 to 8.1)	-0.1 (-0.7 to 0.5)	-0.2 (-6.3 to 5.9)
**Ramsey County**							
HealthRise patients	43	4.7 (1.1 to 17.4)	25.6 (14.5 to 41.0)	10.4 (9.7 to 11.2)	9.0 (8.4 to 9.5)	-1.5 (-2.1 to -0.8)	-11.4 (-16.9 to -5.9)
Comparison patients	72	26.4 (17.4 to 37.9)	29.2 (19.7 to 40.8)	10.0 (9.4 to 10.6)	9.8 (9.2 to 10.4)	-0.2 (-0.8 to 0.5)	2.7 (-4.9 to 10.3)
**Rice County**	** **	** **	** **	** **	** **	** **	** **
HealthRise patients	96	43.8 (34.1 to 53.9)	51.0 (41.0 to 61.0)	8.8 (8.3 to 9.2)	8.5 (8.1 to 9.0)	-0.3 (-0.6 to 0.1)	-1.3 (-4.9 to 2.4)
Comparison patients	296	50.3 (44.6 to 56.0)	54.7 (49.0 to 60.3)	8.6 (8.3 to 8.8)	8.3 (8.1 to 8.6)	-0.2 (-0.5 to 0.0)	0.1 (-2.4 to 2.6)

Across sites, a considerable percentage of hypertension patients with baseline SBP measures exceeding 140 mmHg improved endline levels to below 140 mmHg (Fig 1A); this trend was particularly pronounced for Hennepin and Ramsey. In Hennepin, 76.8% (95% confidence interval: 65.6 to 84.4%) of hypertension patients enrolled in HealthRise recorded endline SBP measures below 140 mmHg and 50.0% (32.3 to 67.3%) of HealthRise patients with hypertension in Ramsey met this threshold. Nonetheless, some percentage of hypertension patients shifted into worse SBP categories by endline: 17.5% in Hennepin, 9.4% in Ramsey, and 13.8% in Rice (Fig 1A; S1 Data).

Sizeable improvements occurred for diabetes patients meeting clinical targets since enrollment (Fig 1B), especially for Ramsey. Compared with baseline, where fewer than 5% of patients with diabetes were meeting treatment targets, 25.6% (14.5 to 41.0%) of Ramsey HealthRise patients with diabetes had A1c levels lower than 8%. Yet many HealthRise patients with diabetes still had A1c levels of 8% or higher by endline across sites: 75.7% in Hennepin, 74.4% in Ramsey, and 49.0% in Rice. A1c category shifts between baseline and endline were especially varied for Hennepin and Rice; for nearly every A1c category at baseline (i.e., < 7%, 7–7.9%, 8–9.9%, ≥10%), some portion of patients moved to one of the other three A1c categories by endline.

Unadjusted and adjusted difference-in-difference model results were nearly identical for the effect of HealthRise (Table 4); accordingly, we report on the adjusted model results here. Overall, HealthRise patients trended toward greater progress in reducing biometric measures and meeting treatment targets than comparison patients; however, these differences were not consistently significant across indicators and sites. For hypertension patients, HealthRise participation was associated with statistically significant SBP reductions relative to comparison patients in Rice (6.9 mmHg decrease [0.9 to 13.0; *p* < 0.05]). Relative to comparison patients, HealthRise participation was also associated with a statistically significant increase in the percentage of hypertension patients meeting treatment targets in Hennepin (27.3 percentage-point rise [9.7 to 45.0; *p* < 0.01]) and Rice (17.1 percentage-point increase [0.9 to 33.4; *p* < 0.05]). In Ramsey, changes in hypertension indicators were not statistically different between the HealthRise and comparison groups, though program participation trended toward improvement: a 10.7 mmHg decrease (-0.3 to 21.8; *p* = 0.057) in SBP and 22.3 percentage-point increase (-0.01 to 44.4 = 5; *p* = 0.054) in meeting treatment targets since baseline.

**Table 4:** Unadjusted and adjusted difference-in-difference regression results, by HealthRise grantee, for hypertension (A) and diabetes (B) patients.

A) Hypertension						
	**Hennepin County**		**Ramsey County**		**Rice County**	
	Coefficient (95% CI)	*p*-value	Coefficient (95% CI)	*p*-value	Coefficient (95% CI)	*p*-value
**Change in systolic blood pressure (mmHg)**					** **	** **
**Unadjusted model**	** **	** **	** **	** **	** **	** **
HealthRise-endline interaction	-5.6 (-11.7 to 0.4)	0.068	-10.7 (-21.7 to 0.2)	0.054	**-6.9 (-12.9 to -0.9)**	**0.024**
**Adjusted model**	** **	** **	** **	** **	** **	** **
HealthRise-endline interaction	-5.6 (-11.7 to 0.5)	0.070	-10.7 (-21.8 to 0.3)	0.057	**-6.9 (-13.0 to -0.9)**	**0.025**
**Age**	** **	** **	** **	** **	** **	** **
< 50 years	-	-	-	-	-	-
≥ 50 years	1.1 (-4.2 to 6.4)	0.677	3.6 (-2.8 to 10.1)	0.266	1.4 (-2.9 to 5.7)	0.531
**Sex**						
Male	-	-	-	-	-	-
Female	-0.3 (-4.6 to 4.1)	0.909	2.6 (-4.3 to 9.6)	0.455	0.5 (-3.2 to 4.2)	0.780
**Duration from baseline to endline**						
< 12 months	-	-	-	-	-	-
≥ 12 months	-0.8 (-8.0 to 6.4)	0.829	-2.0 (-11.3 to 7.2)	0.663	**-9.1 (-14.1 to -4.2)**	**< 0.001**
**Comorbid**						
No (hypertension only)	-	-	-	-	-	-
Yes (hypertension and diabetes)	-5.6 (-11.7 to 0.5)	0.266	-7.4 (-15.1 to 0.3)	0.061	**8.5 (2.5 to 14.5)**	**0.006**
**Change in patients meeting treatment targets (% points)**						
**Unadjusted model**	** **	** **	** **	** **	** **	** **
HealthRise-endline interaction	**27.3 (9.8 to 44.9)**	**0.002**	22.3 (-0.0 to 44.8)	0.051	**17.1 (0.9 to 33.3)**	**0.038**
**Adjusted model**	** **	** **	** **	** **	** **	** **
HealthRise-endline interaction	**27.3 (9.7 to 45.0)**	**0.003**	22.3 (-0.0 to 44.5)	0.054	**17.1 (0.9 to 33.4)**	**0.039**
**Age**						
< 50 years	-	-	-	-	-	-
≥ 50 years	2.9 (-12.7 to 18.6)	0.714	-2.4 (-18.8 to 14.1)	0.777	7.8 (-1.6 to 17.2)	0.102
**Sex**						
Male	-	-	-	-	-	-
Female	0.1 (-9.0 to 11.0)	0.844	-6.5 (-21.4 to 8.4)	0.389	-1.6 (-9.8 to 6.5)	0.697
**Duration from baseline to endline**						
< 12 months	-	-	-	-	-	-
≥ 12 months	6.0 (-12.1 to 24.1)	0.511	5.2 (11.9 to 22.3)	0.546	**21.2 (11.1 to 31.2)**	**< 0.001**
**Comorbid**						
No (hypertension only)	-	-	-	-	-	-
Yes (hypertension and diabetes)	11.0 (-0.4 to 22.4)	0.058	10.3 (-6.9 to 27.4)	0.238	-8.2 (-21.8 to 5.4)	0.236
**B) Diabetes**	** **	** **	** **	** **	** **	** **
	**Hennepin County**		**Ramsey County**		**Rice County**	
	Coefficient (95% CI)	*p*-value	Coefficient (95% CI)	*p*-value	Coefficient (95% CI)	*p*-value
**Change in A1c (%)**						
**Unadjusted model**	** **	** **	** **	** **	** **	** **
HealthRise-endline interaction	-0.4 (-1.5 to 0.8)	0.551	**-1.3 (-2.2 to -0.4)**	**0.007**	-0.03 (-0.5 to 0.4)	0.904
**Adjusted model**	** **	** **	** **	** **	** **	** **
HealthRise-endline interaction	-0.4 (-1.5 to 0.8)	0.556	**-1.3 (-2.3 to -0.4)**	**0.007**	-0.03 (-0.5 to 0.4)	0.905
**Age**						
< 50 years	-	-	-	-	-	-
≥ 50 years	**-1.8 (-3.0 to -0.6)**	**0.006**	0.1 (-0.8 to 1.0)	0.828	-0.1 (-0.6 to 0.4)	0.369
**Sex**						
Male	-	-	-	-	-	-
Female	0.1 (-0.7 to 0.9)	0.833	-0.1 (-0.9 to 0.7)	0.791	-0.1 (-0.5 to 0.3)	0.622
**Duration from baseline to endline**						
< 12 months	-	-	-	-	-	-
≥ 12 months	**-1.5 (-2.8 to -0.1)**	**0.031**	0.2 (-0.8 to 1.1)	0.807	0.2 (-0.2 to -0.6)	0.369
**Comorbid**						
No (diabetes only)	-	-	-	-	-	-
Yes (hypertension and diabetes)	-0.5 (-1.7 to 0.7)	0.417	-0.5 (-1.3 to 0.4)	0.260	0.1 (-0.3 to -0.6)	0.510
**Change in patients meeting treatment targets (% points)**						
**Unadjusted model**	** **	** **	** **	** **	** **	** **
HealthRise-endline interaction	-5.1 (-30.2 to 19.9)	0.684	18.2 (-0.2 to 36.5)	0.052	3.0 (-8.3 to 14.1)	0.612
**Adjusted model**	** **	** **	** **	** **		
HealthRise-endline interaction	-5.1 (-30.5 to 20.2)	0.688	18.2 (-0.4 to 36.7)	0.055	3.0 (-8.4 to 14.2)	0.613
**Age**						
< 50 years	-	-	-	-	-	-
≥ 50 years	9.0 (-10.1 to 28.2)	0.351	4.5 (-8.6 to 17.6)	0.498	-2.6 (-12.5 to 7.2)	0.596
**Sex**						
Male	-	-	-	-	-	-
Female	-7.8 (-24.0 to 8.3)	0.336	-2.1 (-14.5 to 10.3)	0.739	0.8 (-8.2 to 9.8)	0.867
**Duration from baseline to endline**						
< 12 months	-	-	-	-	-	-
≥ 12 months	13.8 (-11.2 to 38.8)	0.274	-0.3 (-14.8 to 14.1)	0.963	-2.7 (-12.4 to 7.0)	0.582
**Comorbid**						
No (diabetes only)	-	-	-	-	-	-
Yes (hypertension and diabetes)	0.9 (-19.6 to 21.5)	0.929	1.9 (-10.5 to 14.3)	0.760	2.9 (-8.4 to 14.2)	0.648

Patient samples included in this analysis are those who met all inclusion critiera, remained enrolled throughout the program (for HealthRise patients), and had at least two biometric readings to reflect potential changes between baseline and endline. Bolded values reflect statistically significant estimates at *p* < 0.05.

Among diabetes patients, HealthRise participation was associated with statistically significant reductions in A1c in Ramsey (1.3 decrease in A1c [0.4–3.2; *p* < 0.01) relative to comparison patients. While the percentage of HealthRise patients meeting treatment targets for diabetes did not statistically differ from that of comparison patients in Ramsey, this indicator trended toward improvement as well (a 18.2 percentage-point increase [-0.4–36.7; *p* = 0.054]).

Sensitivity analyses were conducted adjusting for baseline readings of SBP and A1c to test whether patients experiencing worse clinical profiles at baseline were more likely to experience improvements by endline (S2 Table). Models including these adjustments indicated otherwise, such that not meeting treatment targets at baseline (i.e., < 140 mmHg for SBP; < 8% A1c) was associated with higher average SBP or A1c readings at endline measurement. The estimated effects of HealthRise participation did not change after adjusting for baseline readings, both in terms of continuous baseline measures and binary indicators of meeting treatment targets.

### Qualitative findings

Across HealthRise sites, six main themes emerged for the qualitative data synthesis (Table 5). First, respondents viewed home-based providers as critical to bridging barriers experienced by patients (e.g., linguistic and cultural divides), and clinic-based providers indicated high value in meeting patients beyond clinical settings. Coordination of care and a focus on social determinants of health, such as access to healthier food and nutrition, were highlighted as key program features. Second, program strengths involved learning from HealthRise sites in other countries and enabling many clinical staff to work with in-home providers for the first time. Clinical providers reported improved quality and efficiency in clinical appointments due to having additional details about patient needs from in-home providers. Home visits also enabled providers to connect patients with non-clinical resources (e.g., housing) to support improved outcomes. The theme of perceived program impacts extended program strengths, with providers reporting positive changes in the health and lives of patients and their families. Further, several interviewees emphasized the synergistic effects of pairing CHWs and community paramedics (CPs) within care teams, and reported efforts to adopt home-based provider models by other local organizations because of HealthRise experiences.

**Table 5:** Summary of key themes, components, and quotes from qualitative data synthesized across US HealthRise sites.

HealthRise thematic area components and contexts	Sample thematic quotes
**Theme 1: Key program features**	
• Home-based providers as the cornerstone to HealthRise model, bridging linguistic and cultural divides and gaining valuable new information on home visits	“…through care coordination, communication, and use of frontline health workers…in ways we’ve never been able to before, connect[ing] with families and follow[ing] up with specific patients to help them really understand and manage their chronic disease.”–*Clinic-based provider*
• Emphasis on care coordination and extending care outside the clinic to address social determinants of health	“There are so many hard things about managing diabetes. . .it takes so much time for any patient to fully understand how to put the different parts of diabetes treatment together. . .giving people the time they need to really understand all the components of diabetes control. . .that’s where our CHWs have really been massive assets.”–*Clinic-based provider*
	“If 80% of health happens outside the clinical setting, what are the ways we can foster healthy environments that allow individuals more capacity and agency to focus on these chronic diseases?”–*Administrator*
**Theme 2: Program strengths**	
• Global learning from other HealthRise sites to inform intervention design	“The global aspect is quite unique…utilizing similar strategies in different countries with very different health systems but with a similar population focus and similar workforce approaches…. I’m not aware of other projects that have attempted that across a set of different jurisdictions and landscapes.”–*Policymaker*
• Introduction to the value of home-based providers for many clinic staff	
• Strong relationships built between different types of providers over course of program	“The home visits contributed to more rational use of clinic time…and improved care on my end. From listening to CHWs, I have a better understanding of what’s going on in people’s lives.”–*Clinic-based provider*
• Patients’ receipt of extra support beyond what was typically possible in limited time of clinical appointment	
**Theme 3: Program challenges**	
• Clinical providers lacked familiarity with home-based providers	“We’ve learned that a lot of the hurdle we have to get past is educating other health care providers on what we do…what is a CP and how can we be part of their team and help to better serve their patients. . .the ones who do now understand our role. . .they are our champions, they get so excited. . .we definitely see resistance in the beginning.”–*CP*
• Facility administrators lacked experience managing home-based providers	
• High turnover of staff, both community health workers and management	
• Expectations to improve clinical outcomes in short period of time	“It was too short of a time. . .it took forever to get these communities up and running, get people hired. . .people quit, etc. . .need a longer lifespan than three years. . .to show enough impact to indicate policy change.”—*Policymaker*
• Times lags between patient recruitment/consent and actual program enrollment	
• Patients’ barriers to accessing healthcare (e.g., social determinants of health), as well as provider barriers to effectively accessing patients (e.g., language)	“One of the largest hurdles and barrier to successful implementation was the lack of cohesive patient data systems…you need layers of permission, use agreements, consent, you can’t compare across systems, you can’t look at anybody’s system but your own. . .if somebody could fix that, we could do a lot more good.”—*Administrator*
• Interoperability and data sharing between health systems, across platforms, and among care team (e.g., electronic medical record access for all team members)	
**Theme 4: Perceived program impacts**	
• Information gained during home visits improved the quality and efficiency of clinical appointments	“There’s just a synergy when you combine the two. . .the CP and the medical side and the CHW looking at the social issues. . .it was kind of exponential how much benefit we were able to provide versus just one or the other.”—*CP*
• Pairing of CP and CHW brought together complementary skill sets	
• Home visits helped connect patients with non-clinical resources to improve health	“We have nutritionists and a diabetic educator in the clinic, but patients for a variety of reasons are not always open to meeting with them….to have somebody go to their house and figure out what their specific food interests are and come up with recipes, that was great.”–*Clinic-based provider*
• Positive changes in health and lives of patients and their families	
• Efforts to institutionalize home-based providers as part of care model	
**Theme 5: Recommendations for improvement**	
• Tailor EMR software for care teams that include home-based providers	“EMRs are designed around providers and reimbursements, our model is around holistic care coordination across contexts. The tools we have been using are imperfect, we’re looking for other tools that might be able to plug in to our model better. . .We haven’t yet found the silver bullet.”–*Administrator*
• Determine ideal duration and/or frequency of home visits	
• Offer additional mental health resources to support patients with chronic disease	“We’re aggressively moving into providing more mental health care, plugging it into our model that we’ve perfected during HealthRise…Behavioral health, mental health, and chemical dependency are NCDs, similar to diabetes in that it’s all about what happens in the meantime.”–*Administrator*
• Provide more trainings for home-based providers (e.g. motivational interviewing)	
**Theme 6: Future program directions**	
• Expand use of home-based providers to other patient populations, particularly for other chronic conditions	“I think it should be implemented everywhere…that’s the response we’re getting from our partners, from others in the health care setting…everyone is saying ’CHW! I need 7 of you in my facility!’…everyone has a million questions about how to get it started, set up. . .I definitely think it’s a model to follow.”—C*HW*
• Generate additional evidence establishing the cost-effectiveness of home-based care for chronic conditions	
• Identify sustainable funding for home-based providers	

Common challenges emerged across sites, often relating to new program establishment and incorporation of in-home providers within care teams. For example, some clinical providers showed initial skepticism about the added value of in-home providers, and most administrators did not have prior experience managing CHWs and CPs. Site-specific challenges also occurred; for instance, patient consent for enrollment took a long time during the initial phase of program implementation in Rice, while CHW turnover was an ongoing obstacle for both Hennepin and Ramsey. Data sharing was another pervasive challenge, mainly from poor interoperability and coordination between clinic data systems and electronic medical record (EMR) systems. Providers also expressed frustration with expectations for rapidly improving clinical outcomes, especially given the longstanding challenges in healthcare access and social determinants of health most patients faced.

The fifth theme pertained to recommendations for improvement, many of which stemmed from acknowledged challenges. Such suggestions included prioritizing better communication and coordination among care teams as well as EMR systems that could more seamlessly accommodate patient updates and provider notes from multiple care-team members. Interviewees reported having inadequate clarity on the ideal frequency and length of home visits, an area where efficiencies in resource deployment could be improved. The sixth theme, future program directions, involved many ideas about adapting and expanding HealthRise programming to new locations and conditions (e.g., mental health). Another common thread concerned program sustainability, namely longer-term financing and retaining home-based providers for the HealthRise model.

## Discussion

Increasingly more evidence shows that community-based programs can help underserved communities in the US better access health services, alleviate barriers to care, and improve at least some health behaviors and outcomes. The present study contributes to this evidence base through its prospective evaluation of HealthRise programs implemented within three different Minnesota communities. Relative to comparison patients in Rice County, HealthRise participation was significantly associated with SBP reductions, while the percentage of hypertension patients meeting treatment targets increased at the Hennepin and Rice HealthRise sites. For diabetes, HealthRise patients saw larger A1c declines in at the Ramsey site than comparison patients. Heterogeneous patterns in patient improvements since baseline highlight potential case management challenges among underserved individuals and communities, especially under short program implementation periods. As emphasized by HealthRise care teams, community-based programs show promise for improving NCD care and outcomes for underserved populations; nonetheless, more work is needed to better understand how such programs can be further brought to scale and sustained long-term.

HealthRise participation was related to SBP or A1c decreases and a higher percentage of patients meeting treatment targets at some sites relative to comparison patients. Variations found across sites may be related to the types of specific interventions and activities implemented, as HealthRise programs were meant to be tailored to local contexts and needs [14, 15]. For instance, one component of the Ramsey HealthRise program was to implement nutrition-focused interventions in both English and Spanish, as English language barriers were identified as a key challenge for patients served by the Ramsey site (Table 1) and 50% of Ramsey HealthRise participants identified as Hispanic or Latinx at enrollment (S1 Table). Alternatively, such variation may be associated with the relative health status of each population at baseline and thus potential for future improvement. At the Ramsey site, HealthRise patients with diabetes averaged 11.4% reductions in A1c and 9.4% declines in SBP by endline; yet these patients also began HealthRise with highest risk profiles, averaging 150 mmHg SBP and 10% A1c at baseline. Sensitivity analyses adjusting for baseline readings found no changes in the effects associated with HealthRise participation (S2 Table), whereas not meeting treatment targets at baseline measurement was associated with increases in SBP or A1c by endline. In combination, these results suggest that, all else being equal, patients with higher risk profiles may, on average, experience worsening indicators over time–and that community-based interventions such the HealthRise program could play a role in lessening or reversing such progression.

As found in past studies [5–7], several factors may have contributed to the observable effects of HealthRise. These included focusing on specific barriers patients faced in each community (e.g., home-based care provided by CHW and CP teams); maintaining small patient loads, enabling more individualized attention and tailoring of visit frequency to patient need; and explicitly providing non-medical support, such as health education and community resources like transportation. Further, the overall positive views of HealthRise by grantees and care teams alike may have contributed to the program’s effects. Despite challenges during earlier stages of program implementation (e.g., recruiting and retaining CHWs, ensuring adequate access to EMRs), providers voiced valuing home-based health workers and were eager to expand this model of care. In combination, these factors may have set the foundation for HealthRise’s impact for underserved patients with hypertension and diabetes in the US.

Amid such promising findings, however, important challenges remained for each site and for broader applications of the HealthRise model elsewhere. Across sites, some proportion of HealthRise patients failed to meet clinical targets for hypertension or diabetes at both time points–and concerningly, some percentage moved from being below biometric thresholds at baseline to exceeding them at endline. These patterns underscore the complexity of effectively managing chronic conditions like hypertension and diabetes, especially in environments where patients face compounding barriers to medical care and health-promoting behavior (e.g., limited options for nutritious food, minimal time for exercise amid job and family demands, inferior access to adequate transportation and housing). CHWs and integrated care teams may be able to mitigate some of these obstacles and better support patients’ medical needs, a critical step in addressing deep-seeded health disparities; however, in the absence of more macro-level socioeconomic policies and health system investments to support underserved patients in the US, many community-based programs will continue facing need and demand that far exceeds their limited capacities and resources. As laid bare by COVID-19, the health challenges underserved communities experience do not begin and end at clinics: rather, they stem from and are exacerbated by structural inequalities that require intervention and engagement well beyond the formal health system [20, 21] CHWs can provide a vital role in at least overcoming some of these access and sociocultural barriers, ranging from house-based visits made by CHWs fluent in patients’ native languages [3] to connecting patients with services that can facilitate better overall care. Nevertheless, the potential benefits of encouraging health programs can be easily blunted if actors–and actions–beyond the immediate health system are not also actively addressing fundamental drivers of health disparities.

### Limitations

This study is subject to several limitations. First, small program sizes and thus study samples at each HealthRise site likely affected the degree to which potential program impact could be detected and conclusively attributed to HealthRise participation. This is particularly true for site-condition combinations in which very few patients were prevalent cases at baseline and had at least two biometric readings within the program implementation period (e.g., 37 HealthRise patients with diabetes in at the Hennepin site, 32 HealthRise patients with hypertension in Ramsey). The inclusion or exclusion of even a few patients for several site-condition groupings could shift effect sizes and statistical significance estimated by the difference-in-difference models.

Second, comparison groups were constructed retrospectively based on available patient record information and were not selected by random assignment. While efforts were made to ensure that comparison patient data were chosen to generally represent individuals who would have been eligible for HealthRise enrollment, they may have differed from individuals who enrolled.

Third, only patients who remained enrolled at endline were included in the present study; by taking this ‘as treated’ analytic approach, which provides insights into program effects closer to full adherence, these patients may not represent all potential target populations for HealthRise interventions and results may be positively biased. For instance, relatively high rates of program withdrawal at some sites could have led to a bias toward healthier patients remaining in the program (i.e., sicker patients may not go into the clinic). However, due to the home-based care model espoused by HealthRise, it is equally possible that patients who did not withdraw were less healthy and stayed enrolled because HealthRise offered important access to services, like home visits, otherwise unavailable to them.

Fourth, single biometric readings comprised baseline and endline measures, as well as patients meeting clinical targets at each time period; subsequently, analyses could be sensitive to outliers in patient records, particularly given the relatively small sample sizes for each site. If more readings could have informed baseline and endline indicators, it is possible patient-level patterns could have differed from what observed on the basis of single readings.

Fifth, medication data were not available and thus we were unable to assess the full cascade of care for hypertension and diabetes case management. Medication adherence may have been important factor for patients who either failed to see improvements in clinical indicators or experienced worsening outcomes. Furthermore, additional information on risk factors or health behaviors (e.g., smoking status, alcohol consumption) that may have affected patient-level outcomes were not available and thus could not be accounted for.

Sixth, due to the small sample sizes for each site, we could not further analyze the potential effects of visit frequency (and thus approximate dose-response) or intensity of intervention exposure on patient outcomes. This is further complicated by issues related to endogeneity, such that patients with worse clinical profiles and thus greater need are likely to receive more frequent visits by CHWs or care teams.

Seventh, small sample size and inconsistent data availability limited our ability to conduct more disaggregated analyses by reported sex, race or ethnicity, income, and other potentially important factors (e.g., primary language) for understanding how community-based health interventions can promote greater equity for underserved communities. Descriptive statistics imply that there could be differential disease prevalence by reported race or ethnicity (S1 Table): for instance, at the Rice County site, a comparatively smaller percentage of HealthRise patients identifying as Black or African American had both hypertension and diabetes at enrollment (7.0%, *n* = 4 of 57 total patients with both conditions) than the overall percentage of patients identifying as Black or African American had (10.2%, *n* = 16 out 157 total patients). Yet based on such small samples, formal analyses comparing prevalence by reported race or ethnicity could yield inconclusive–or worse, potentially spurious or unrepresentative–results. Combining different reported race or ethnicity groups is one option often used to mitigate small-sample size issues, though to doing so risks minimizing the experiences of a given racial or ethnicity group [22]. For the present study, we ultimately determined that further disaggregation posed more potential analytic harms or opportunities for misinterpretation than possible overall benefits; in doing so, however, we recognize its substantive limitation for the present study.

## Conclusions

With its focus on community-based health programs and improving NCD care for underserved populations, HealthRise showed some positive effects for hypertension and diabetes patients in Hennepin, Ramsey, and Rice counties. Provider experiences indicated enthusiasm for expanding the home-based model of care for NCDs, though the resource requirements–as well feasibility–to sustain impact at a larger scale remain unknown. While community-based NCD interventions show promise for overcoming barriers to effective care for hypertension and diabetes among underserved populations, continued monitoring and robust evaluations of local impact are vital to ensuring maximum benefit for individuals with the greatest need.

## Supporting information

S1 Table Descriptive statistics for HealthRise patients, by site, across all patients (A) and by prevalent case of hypertension, diabetes, or both conditions (B). Percentages are reported in terms of patients meeting inclusion criteria for (A) and patients with each condition for (B). All counts and percentages are directly calculated from de-identified patient data.
(PDF)

S2 Table Sensitivity analyses adjusting for baseline measures of systolic blood pressure (SBP) and A1c for difference-in-difference regression results, by HealthRise site, for hypertension (A) and diabetes (B) patients. Patient samples included in this analysis are those who met all inclusion criteria, remained enrolled throughout the program (for HealthRise patients), and had at least two biometric readings to reflect potential changes between baseline and endline. Bolded values reflect statistically significant estimates at p < 0.05.
(PDF)

S1 Data Percentage of HealthRise patients meeting hypertension and diabetes disease severity categories between baseline and endline, by site. (XLSX)

S2 Data (CSV)
