# Supplementary material for: Assessing the impact of community-based interventions on hypertension and diabetes management in three Minnesota communities: Findings from the prospective evaluation of US HealthRise programs
Source: PLoS One. 2023 Feb 27;18(2):e0279230. doi: 10.1371/journal.pone.0279230 (PMC9970068; doi:10.1371/journal.pone.0279230)
Supplement: S1 Table — Percentages are reported in terms of patients meeting inclusion criteria for (A) and patients with each condition for (B). All counts and percentages are directly calculated from de-identified patient data. (PDF) [file pone.0279230.s001.pdf]

S1 Table. Descriptive statistics for HealthRise patients, by site, across all patients (A) and by prevalent case of hypertension, diabetes, or both conditions (B). Percentages are reported in terms of patients meeting inclusion criteria for (A) and patients with each condition for (B). All counts and percentages are directly calculated from de-identified patient data.

A)

HealthRise patient information	Hennepin County		Ramsey County		Rice County	
	Baseline (%)	Endline (%)	Baseline (%)	Endline (%)	Baseline (%)	Endline (%)
**Patients meeting inclusion criteria**	**114**	**95**	**77**	**52**	**157**	**152**
Patients with hypertension (diagnosed, or ≥ 140 SBP or ≥ 90 DSP)	102 (89.5%)	86 (90.5%)	49 (63.6%)	36 (69.2%)	86 (54.8%)	88 (57.9%)
Patients with diabetes (diagnosed, or ≥ 6.5% A1c)	94 (82.5%)	77 (81.1%)	72 (93.5%)	48 (92.3%)	128 (82.5%)	125 (82.2%)
Patients with hypertension and diabetes (diagnosed, or per readings)	82 (71.9%)	68 (71.6%)	44 (57.1%)	32 (61.5%)	57 (29.9%)	61 (40.1%)
**Demographic information** (*per enrollment data*)						
**Age**						
< 50 years	41 (36.0%)	36 (37.9%)	17 (22.1%)	12 (23.1%)	69 (43.9%)	67 (44.1%)
≥ 50 years	73 (64.0%)	59 (62.1%)	60 (77.9%)	40 (76.9%)	88 (56.1%)	85 (55.9%)
**Reported sex**						
Female	62 (54.4%)	55 (57.9%)	42 (54.5%)	27 (51.9%)	84 (53.5%)	81 (53.3%)
Male	52 (45.6%)	40 (42.1%)	35 (45.5%)	25 (48.1%)	73 (46.5%)	71 (46.7%)
**Reported race/ethnicity**						
American Indian/Indigenous peoples	-	-	1 (1.3%)	1 (1.9%)	-	-
Asian	4 (3.5%)	2 (2.1%)	1 (1.3%)	1 (1.9%)	-	-
Black or African American	68 (59.6%)	59 (62.1%)	16 (20.8%)	7 (13.7%)	16 (10.2%)	15 (9.9%)
Hispanic or Latinx (all races)	2 (1.8%)	2 (2.1%)	41 (53.3%)	33 (63.5%)	134 (85.4%)	130 (85.5%)
Multiracial	-	-	1 (1.3%)	0 (0%)	-	-
White	39 (34.2%)	32 (33.7%)	17 (22.1%)	10 (19.2%)	7 (4.5%)	7 (4.6%)
Not reported	1 (0.9%)	0 (0%)	-	-	-	-

B)

HealthRise patient clinical profiles, disaggregated by demographic information	Hennepin County		Ramsey County		Rice County	
	Baseline (%)	Endline (%)	Baseline (%)	Endline (%)	Baseline (%)	Endline (%)
**Patients with hypertension (diagnosed, or ≥ 140 SBP or ≥ 90 DSP)** - *irrespective of diabetes diagnosis*	**102**	**86**	**49**	**36**	**86**	**88**
**Age**						
< 50 years	35 (34.3%)	31 (36.0%)	8 (16.3%)	7 (19.4%)	31 (36.0%)	31 (35.2%)
≥ 50 years	67 (65.7%)	55 (64.0%)	41 (83.7%)	29 (80.6%)	55 (64.0%)	57 (64.8%)
**Reported sex**						
Female	57 (55.9%)	50 (58.1%)	28 (55.6%)	20 (51.2%)	44 (51.2%)	45 (51.1%)
Male	45 (44.1%)	36 (41.9%)	21 (42.9%)	16 (44.4%)	42 (48.8%)	43 (48.9%)
**Reported race/ethinicity**						
American Indian/Indigenous peoples	-	-	1 (2.0%)	1 (2.8%)	-	-
Asian	4 (3.9%)	2 (2.3%)	1 (2.0%)	1 (2.8%)	-	-
Black or African American	61 (59.8%)	55 (64.0%)	16 (32.7%)	7 (19.4%)	14 (16.3%)	13 (14.8%)
Hispanic or Latinx (all races)	2 (2.0%)	2 (2.3%)	18 (36.7%)	19 (52.8%)	66 (76.7%)	68 (77.3%)
Multiracial	-	-	1 (2.0%)	0 (0.0%)	-	-
White	34 (33.3%)	27 (31.4%)	12 (24.5%)	8 (22.2%)	6 (7.0%)	7 (8.0%)
Not reported	1 (1.0%)	0 (0.0%)	-	-	-	-
**Patients with diabetes (diagnosed, or ≥ 6.5% A1c)** - *irrespective of hypertension diagnosis*	**94**	**77**	**72**	**48**	**128**	**125**
**Age**						
< 50 years	32 (34.0%)	27 (35.1%)	16 (22.2%)	11 (22.9%)	56 (43.8%)	55 (44.0%)
≥ 50 years	62 (66.0%)	50 (64.9%)	56 (77.8%)	37 (77.1%)	72 (56.3%)	70 (56.0%)
**Reported sex**						
Female	47 (50.0%)	40 (51.9%)	39 (54.2%)	25 (52.1%)	71 (55.5%)	69 (55.2%)
Male	47 (50.0%)	37 (48.1%)	33 (45.8%)	23 (47.9%)	57 (44.5%)	56 (44.8%)
**Reported race/ethinicity**						
American Indian/Indigenous peoples	-	-	1 (1.4%)	1 (2.1%)	-	-
Asian	3 (3.2%)	2 (2.6%)	1 (1.4%)	1 (2.1%)	-	-
Black or African American	55 (58.5%)	46 (59.7%)	15 (20.8%)	6 (12.5%)	6 (4.7%)	6 (4.8%)
Hispanic or Latinx (all races)	2 (2.1%)	2 (2.6%)	40 (55.6%)	33 (68.8%)	117 (91.4%)	114 (91.2%)
Multiracial	-	-	1 (1.4%)	0 (0.0%)	-	-
White	33 (35.1%)	27 (35.1%)	14 (19.4%)	7 (14.6%)	5 (3.9%)	5 (4.0%)
Not reported	1 (1.1%)	0 (0.0%)	-	-	-	-
**Patients with hypertension and diabetes (diagnosed, or per readings: ≥ 140 SBP or ≥ 90 DSP, and ≥ 6.5% A1c)**	**82**	**68**	**44**	**32**	**57**	**61**
**Age**						
< 50 years	26 (31.7%)	22 (32.4%)	7 (15.9%)	6 (18.8%)	18 (31.6%)	19 (31.1%)
≥ 50 years	56 (68.3%)	46 (67.6%)	37 (84.1%)	26 (81.3%)	39 (68.4%)	42 (68.9%)
**Reported sex**						
Female	42 (51.2%)	35 (51.5%)	25 (56.8%)	18 (56.3%)	31 (54.4%)	33 (54.1%)
Male	40 (48.8%)	33 (48.5%)	19 (43.2%)	14 (43.8%)	26 (45.6%)	28 (45.9%)
**Reported race/ethinicity**						
American Indian/Indigenous peoples	-	-	1 (2.3%)	1 (3.1%)	-	-
Asian	3 (3.7%)	2 (2.9%)	1 (2.3%)	1 (3.1%)	-	-
Black or African American	48 (58.5%)	42 (61.8%)	15 (34.1%)	6 (18.8%)	4 (7.0%)	4 (6.6%)
Hispanic or Latinx (all races)	2 (2.4%)	2 (2.9%)	17 (38.6%)	19 (59.4%)	49 (86.0%)	52 (85.2%)
Multiracial	-	-	1 (2.3%)	0 (0.0%)	-	-
White	28 (34.1%)	22 (32.4%)	9	5	4 (7.0%)	5 (8.2%)
Not reported	1 (1.2%)	0 (0.0%)	-	-	-	-
