# Supplementary material for: Assessing the impact of community-based interventions on hypertension and diabetes management in three Minnesota communities: Findings from the prospective evaluation of US HealthRise programs
Source: PLoS One. 2023 Feb 27;18(2):e0279230. doi: 10.1371/journal.pone.0279230 (PMC9970068; doi:10.1371/journal.pone.0279230)
Supplement: S2 Table — Patient samples included in this analysis are those who met all inclusion criteria, remained enrolled throughout the program (for HealthRise patients), and had at least two biometric readings to reflect potential changes between baseline and endline. Bolded values reflect statistically significant estimates at p < 0.05. (PDF) [file pone.0279230.s002.pdf]

**S3 Table. Sensitivity analyses adjusting for baseline measures of systolic blood pressure (SBP) and A1c for difference-in-difference regression results, by HealthRise site, for hypertension (A) and diabetes (B) patients.** Patient samples included in this analysis are those who met all inclusion criteria, remained enrolled throughout the program (for HealthRise patients), and had at least two biometric readings to reflect potential changes between baseline and endline. Bolded values reflect statistically significant estimates at $p < 0.05$.

**A) Hypertension**

| | Hennepin County | | Ramsey County | | Rice County | |
|---|---|---|---|---|---|---|
| | Coefficient (95% CI) | *p* -value | Coefficient (95% CI) | *p* -value | Coefficient (95% CI) | *p* -value |
| **Change in systolic blood pressure (SBP, mmHg)** | | | | | | |
| **Unadjusted model (original)** | | | | | | |
| HealthRise-endline interaction | -5.6 (-11.7 to 0.4) | 0.068 | -10.7 (-21.7 to 0.2) | 0.054 | **-6.9 (-12.9 to -0.9)** | **0.024** |
| **Unadjusted model + adjusting for baseline SBP (mmHg)** | | | | | | |
| HealthRise-endline interaction | -5.6 (-11.7 to 0.4) | 0.068 | -10.7 (-21.7 to 0.2) | 0.055 | **-6.9 (-12.9 to -0.9)** | **0.024** |
| Baseline SBP (mmHg) | **0.6 (0.6 to 0.7)** | **< 0.001** | **0.7 (0.6 to 0.8)** | **< 0.001** | **0.7 (0.6 to 0.8)** | **< 0.001** |
| **Unadjusted model + adjusting for baseline SBP target** | | | | | | |
| HealthRise-endline interaction | -5.6 (-11.7 to 0.4) | 0.068 | -10.7 (-21.7 to 0.2) | 0.055 | **-6.9 (-12.9 to -0.9)** | **0.024** |
| Baseline SBP meets clinical target | | | | | | |
| Yes (< 140 mmHg) | - | - | - | - | - | - |
| No (≥ 140 mmHg) | **15.8 (12.2 to 19.4)** | **< 0.001** | **24.3 (18.2 to 30.3)** | **< 0.001** | **20.8 (16.8 to 24.8)** | **< 0.001** |
| **Adjusted model (original)** | | | | | | |
| HealthRise-endline interaction | -5.6 (-11.7 to 0.5) | 0.070 | -10.7 (-21.8 to 0.3) | 0.057 | **-6.9 (-13.0 to -0.9)** | **0.025** |
| Age | | | | | | |
| < 50 years | - | - | - | - | - | - |
| ≥ 50 years | 1.1 (-4.2 to 6.4) | 0.677 | 3.6 (-2.8 to 10.1) | 0.266 | 1.4 (-2.9 to 5.7) | 0.531 |
| Sex | | | | | | |
| Male | - | - | - | - | - | - |
| Female | -0.3 (-4.6 to 4.1) | 0.909 | 2.6 (-4.3 to 9.6) | 0.455 | 0.5 (-3.2 to 4.2) | 0.780 |
| Duration from baseline to endline | | | | | | |
| < 12 months | - | - | - | - | - | - |
| ≥ 12 months | -0.8 (-8.0 to 6.4) | 0.829 | -2.0 (-11.3 to 7.2) | 0.663 | **-9.1 (-14.1 to -4.2)** | **< 0.001** |
| Comorbid | | | | | | |
| No (hypertension only) | - | - | - | - | - | - |
| Yes (hypertension and diabetes) | -5.6 (-11.7 to 0.5) | 0.266 | -7.4 (-15.1 to 0.3) | 0.061 | **8.5 (2.5 to 14.5)** | **0.006** |
| **Adjusted model (+ adjusting for baseline SBP [mmHg])** | | | | | | |
| HealthRise-endline interaction | -5.6 (-11.7 to 0.5) | 0.070 | -10.7 (-21.8 to 0.3) | 0.058 | **-6.9 (-13.0 to -0.9)** | **0.025** |
| Baseline SBP (mmHg) | **0.6 (0.6 to 0.7)** | **< 0.001** | **0.7 (0.6 to 0.8)** | **< 0.001** | **0.7 (0.6 to 0.8)** | **< 0.001** |
| Age | | | | | | |
| < 50 years | - | - | - | - | - | - |
| ≥ 50 years | -2.1 (-4.8 to 0.5) | 0.113 | **5.5 (0.8 to 10.2)** | **0.022** | -0.8 (-3.7 to 2.0) | 0.559 |
| Sex | | | | | | |
| Male | - | - | - | - | - | - |
| Female | 0.7 (-1.6 to 3.1) | 0.551 | -0.7 (-5.5 to 4.1) | 0.771 | 0.4 (-1.7 to 2.5) | 0.707 |
| Duration from baseline to endline | | | | | | |
| < 12 months | - | - | - | - | - | - |
| ≥ 12 months | 1.1 (-2.2 to 4.4) | 0.515 | -1.2 (-7.3 to 4.9) | 0.695 | -1.2 (-3.5 to 1.1) | 0.313 |
| Comorbid | | | | | | |
| No (hypertension only) | - | - | - | - | - | - |
| Yes (hypertension and diabetes) | -0.07 (-2.6 to 2.4) | 0.957 | -2.3 (-7.9 to 3.3) | 0.413 | **5.5 (1.1 to 9.9)** | **0.014** |
| **Adjusted model (+ adjusting for baseline SBP target)** | | | | | | |
| HealthRise-endline interaction | -5.6 (-11.7 to 0.5) | 0.070 | -10.7 (-21.8 to 0.3) | 0.058 | **-6.9 (-12.9 to -0.9)** | **0.024** |
| Baseline SBP meets clinical target | | | | | | |
| Yes (< 140 mmHg) | - | - | - | - | - | - |
| No (≥ 140 mmHg) | **15.8 (12.2 to 19.3)** | **< 0.001** | **24.3 (18.2 to 30.3)** | **< 0.001** | **20.1 (16.4 to 23.8)** | **< 0.001** |
| Age | | | | | | |
| < 50 years | - | - | - | - | - | - |
| ≥ 50 years | 0.4 (-3.6 to 4.4) | 0.834 | **5.8 (0.04 to 11.6)** | **0.048** | 3.2 (-0.6 to 6.8) | 0.096 |
| Sex | | | | | | |
| Male | - | - | - | - | - | - |
| Female | 0.5 (-3.2 to 4.2) | 0.787 | -0.8 (-6.4 to 4.8) | 0.773 | 0.2 (-2.9 to 3.2) | 0.918 |
| Duration from baseline to endline | | | | | | |
| < 12 months | - | - | - | - | - | - |
| ≥ 12 months | 0.2 (-5.1 to 5.5) | 0.942 | -2.3 (-10.2 to 5.6) | 0.567 | -3.4 (-7.0 to 0.2) | 0.068 |
| Comorbid | | | | | | |
| No (hypertension only) | - | - | - | - | - | - |
| Yes (hypertension and diabetes) | -0.6 (-4.4 to 3.2) | 0.760 | -3.9 (-11.0 to 3.2) | 0.273 | **7.3 (1.7 to 12.9)** | **0.011** |

**B) Diabetes**

| | Hennepin County | | Ramsey County | | Rice County | |
|---|---|---|---|---|---|---|
| | Coefficient (95% CI) | *p* -value | Coefficient (95% CI) | *p* -value | Coefficient (95% CI) | *p* -value |
| **Change in A1c (%)** | | | | | | |
| **Unadjusted model** | | | | | | |
| HealthRise-endline interaction | -0.4 (-1.5 to 0.8) | 0.551 | **-1.3 (-2.2 to -0.4)** | **0.007** | -0.03 (-0.5 to 0.4) | 0.904 |
| **Unadjusted model + adjusting for baseline A1c (%)** | | | | | | |
| HealthRise-endline interaction | -0.3 (-1.5 to 0.8) | 0.552 | **-1.3 (-2.3 to -0.4)** | **0.007** | -0.03 (-0.5 to 0.4) | 0.904 |
| Baseline A1c (%) | **0.7 (0.5 to 0.9)** | **< 0.001** | **0.7 (0.6 to 0.8)** | **< 0.001** | **0.8 (0.7 to 0.8)** | **< 0.001** |
| **Unadjusted model + adjusting for baseline A1c target** | | | | | | |
| HealthRise-endline interaction | -0.3 (-1.5 to 0.8) | 0.552 | **-1.3 (-2.3 to -0.4)** | **0.007** | -0.03 (-0.5 to 0.4) | 0.904 |
| Baseline A1c meets clinical target | | | | | | |
| Yes (< 8.0%) | - | - | - | - | - | - |
| No (≥ 8.0%) | **2.3 (1.4 to 3.2)** | **< 0.001** | **3.0 (2.3 to 3.8)** | **< 0.001** | **3.0 (2.7 to 3.2)** | **< 0.001** |
| **Adjusted model** | | | | | | |
| HealthRise-endline interaction | -0.4 (-1.5 to 0.8) | 0.556 | **-1.3 (-2.3 to -0.4)** | **0.007** | -0.03 (-0.5 to 0.4) | 0.905 |
| Age | | | | | | |
| < 50 years | - | - | - | - | - | - |
| ≥ 50 years | **-1.8 (-3.0 to -0.6)** | **0.006** | 0.1 (-0.8 to 1.0) | 0.828 | -0.1 (-0.6 to 0.4) | 0.369 |
| Sex | | | | | | |
| Male | - | - | - | - | - | - |
| Female | 0.1 (-0.7 to 0.9) | 0.833 | -0.1 (-0.9 to 0.7) | 0.791 | -0.1 (-0.5 to 0.3) | 0.622 |
| Duration from baseline to endline | | | | | | |
| < 12 months | - | - | - | - | - | - |
| ≥ 12 months | **-1.5 (-2.8 to -0.1)** | **0.031** | 0.2 (-0.8 to 1.1) | 0.807 | 0.2 (-0.2 to -0.6) | 0.369 |
| Comorbid | | | | | | |
| No (diabetes only) | - | - | - | - | - | - |
| Yes (hypertension and diabetes) | -0.5 (-1.7 to 0.7) | 0.417 | -0.5 (-1.3 to 0.4) | 0.260 | 0.1 (-0.3 to -0.6) | 0.510 |
| **Adjusted model (+ adjusting for baseline A1c [%])** | | | | | | |
| HealthRise-endline interaction | -0.3 (-1.5 to 0.8) | 0.558 | **-1.3 (-2.3 to -0.4)** | **0.007** | -0.03 (-0.5 to 0.4) | 0.905 |
| Baseline A1c (%) | **0.7 (0.5 to 0.8)** | **< 0.001** | **0.7 (0.6 to 0.8)** | **< 0.001** | **0.8 (0.7 to 0.8)** | **< 0.001** |
| Age | | | | | | |
| < 50 years | - | - | - | - | - | - |
| ≥ 50 years | -0.9 (-1.8 to 0.06) | 0.067 | 0.01 (-0.4 to 0.4) | 0.972 | 0.01 (-0.2 to 0.2) | 0.955 |
| Sex | | | | | | |
| Male | - | - | - | - | - | - |
| Female | -0.05 (-0.6 to 0.5) | 0.852 | 0.06 (-0.3 to 0.5) | 0.749 | 0.01 (-0.2 to 0.2) | 0.912 |
| Duration from baseline to endline | | | | | | |
| < 12 months | - | - | - | - | - | - |
| ≥ 12 months | -0.7 (-1.6 to 0.2) | 0.122 | 0.4 (-0.1 to 0.9) | 0.098 | **0.2 (0.02 to 0.4)** | **0.027** |
| Comorbid | | | | | | |
| No (diabetes only) | - | - | - | - | - | - |
| Yes (hypertension and diabetes) | -0.4 (-1.4 to 0.5) | 0.372 | 0.1 (-0.3 to 0.5) | 0.661 | 0.01 (-0.2 to 0.2) | 0.927 |
| **Adjusted model (+ adjusting for baseline A1c target)** | | | | | | |
| HealthRise-endline interaction | -0.3 (-1.5 to 0.8) | 0.558 | **-1.3 (-2.3 to -0.4)** | **0.007** | -0.03 (-0.5 to 0.4) | 0.905 |
| Baseline A1c meets clinical target | | | | | | |
| Yes (< 8.0%) | - | - | - | - | - | - |
| No (≥ 8.0%) | **2.0 (1.2 to 2.8)** | **< 0.001** | **3.1 (2.4 to 3.9)** | **< 0.001** | **3.0 (2.7 to 3.2)** | **< 0.001** |
| Age | | | | | | |
| < 50 years | - | - | - | - | - | - |
| ≥ 50 years | **-1.5 (-2.7 to -0.3)** | **0.015** | 0.3 (-0.5 to 1.1) | 0.444 | -0.2 (-0.5 to 0.1) | 0.274 |
| Sex | | | | | | |
| Male | - | - | - | - | - | - |
| Female | 0.04 (-0.7 to 0.7) | 0.909 | -0.3 (-0.9 to 0.4) | 0.436 | -0.08 (-0.4 to 0.2) | 0.560 |
| Duration from baseline to endline | | | | | | |
| < 12 months | - | - | - | - | - | - |
| ≥ 12 months | **-1.2 (-2.1 to -0.05)** | **0.041** | 0.3 (-0.5 to 1.1) | 0.402 | **0.3 (0.01 to 0.6)** | **0.039** |
| Comorbid | | | | | | |
| No (diabetes only) | - | - | - | - | - | - |
| Yes (hypertension and diabetes) | -0.3 (-1.4 to 0.8) | 0.583 | -0.1 (-0.9 to 0.7) | 0.786 | -0.01 (-0.3 to 0.3) | 0.938 |
